# Yesterday’s Doctors: The Human Aspects of Medical Education in Britain, 1957–93

**DOI:** 10.1017/mdh.2016.100

**Published:** 2017-01

**Authors:** Victoria Bates

**Affiliations:** Department of History, School of Humanities, University of Bristol, 11 Woodland Road, BS8 1TB, BristolUK

**Keywords:** Medical education, Human aspects of medicine, Social medicine, Ethics, Communication skills, General practice

## Abstract

In the wake of the Second World War there was a movement to counterbalance the apparently increasingly technical nature of medical education. These reforms sought a more holistic model of care and to put people – rather than diseases – back at the centre of medical practice and medical education. This article shows that students often drove the early stages of education reform. Their innovations focused on relationships between doctors and their communities, and often took the form of informal discussions about medical ethics and the social dimensions of primary care. Medical schools began to pursue ‘humanistic’ education more formally from the 1980s onwards, particularly within the context of general practice curricula and with a focus on individual doctor–patient relationships. Overall from the 1950s to the 1990s there was a broad shift in discussions of the human aspects of medical education: from interest in patient communities to individuals; from social concerns to personal characteristics; and from the relatively abstract to the measurable and instrumental. There was no clear shift from ‘less’ to ‘more’ humanistic education, but rather a shift in the perceived goals of integrating human aspects of medical education. The human aspects of medicine show the importance of student activism in driving forward community and ethical medicine, and provide an important backdrop to the rise of competencies within general undergraduate education.

In 1914 *Punch* printed an image entitled ‘Candidate for medical degree being examined in the subject of “Bedside Manner”’.^1^ In the cartoon a trainee doctor bestows attention upon a limp human-sized doll, while four stern superiors assess his skills. The graphic satire of this image highlights the scientific ethos of medical education at the time, in which the process of learning to care for patients was apparently secondary to learning to cure them. However, in the late twentieth century medical students and educators demonstrated a renewed interest in the promotion of ‘human’ values in science and in relationships – both collective and individual – between doctors and their patients. This article examines such human aspects of medical education in British medical schools in the late twentieth century. It focuses on the period between 1957 and 1993, in which concerns about ‘dehumanisation’ overlapped with General Medical Council reform that liberalised medical education.^2^

The article examines a wide range of different practices and labels, including terms often used interchangeably in the period such as ‘humanitarian’, ‘humanism’, ‘humanistic’ and ‘humanise’. It addresses these different strands of human aspects of medical education because they were so entangled and – despite differences between their underlying meanings – had a collective symbolic strength and drew upon shared concerns. It also uses the term ‘human *aspects*…’ in order to avoid implicit assumptions that everything outside ‘humanistic medicine’ is ‘dehumanised’.^3^ In itself the term makes no claims about where ‘human aspects’ lie, but this article indicates that students and educators often located the ‘human’ in a range of sites beyond the biomedical and technological.

Calls for a more human approach to medical education were particularly significant, albeit not entirely new, in the second half of the twentieth century. The Belgian historian George Sarton’s work on New Humanism in the early twentieth century had emphasised the value of multidisciplinary education for ‘humanism’ in science.^4^ The notorious Flexner report (1910), which promoted a ‘scientific’ fact-based education in the United States and was widely known in the United Kingdom, had actually highlighted the value of the humanities for the sciences and had much in common with New Humanism.^5^ However, Edmund Pellegrino notes that Flexner’s work, despite its original goals, became a post-war ‘rhetorical device’ to represent reductionist scientism and to promote change.^6^ This late twentieth-century backlash against Flexner was indicative of growing fears, irrespective of whether they were justified, about the threat that ‘scientific’ education posed to the ‘human’ aspects of medicine. Despite the importance of this period both for medical education and for concerns about the ‘human’ within medicine, it has received little historiographical attention. Many histories of medical education in the UK have focused on the period before the mid-twentieth century.^7^ Others have covered longer periods up to including the late twentieth century, but have focused on particular institutions or the growth of specific subjects.^8^

This article reframes such histories by looking through the lens of a concept, the ‘human’ in medical education, rather than a particular subject or single place. In exploring this subject it touches upon histories of primary care, medical ethics, social medicine and doctor–patient relations in medical education but is not a comprehensive history of any single subject or discipline. Instead, the article explores the varied educational implications of growing dissatisfaction with medicine perceived to prioritise biomedical science and technology without sufficient attention to the patient. It shows that there were varied responses to apparent ‘dehumanisation’, including some resistance to such claims, but that there were some general trends also. Firstly, students were often the early initiators of changes in the culture of medical schools. Secondly, there was a general shift from a community to an individual model of the ‘human’ patient over the late twentieth century. Finally, despite their differences, students and educators alike focused on general practice as a key site of building human relationships at both community and individual levels.

The following analysis begins with a discussion of the teaching of social medicine (human lives) and medical or scientific ethics (human rights) in the 1960s and 1970s. It examines medical students’ initiatives and interest in such subjects, which generally predated medical schools’ curriculum changes. It then considers how doctors’ communication skills (human relationships) and doctors’ attitudes (humanity) later became central to the teaching of ‘humanism’ or ‘humanitarianism’, particularly when medical schools introduced undergraduate courses in the human aspects of medical education. Throughout, alongside discussion of international literature and some national recommendations, the article draws upon some examples from undergraduate courses at medical schools in London, Manchester and Aberdeen.^9^ The three locations provide, in theory, an opportunity to explore trends resulting from significantly different medical school cultures: the systems found in London, Scotland and in all other UK medical schools.^10^

However, this research actually indicates that early forays into teaching the human aspects of medical education were the result of individual or small group action rather than that of medical school cultures, of administration or the result of curriculum structures. London had some of the earliest initiatives, but seemingly as a product of student groups (London Medical Group) and key individuals (such as John Ellis) rather than from any inherent quality of the London medical schools; indeed, some London schools made very few changes while others were crucial to promoting the human aspects of medical education. Structurally, these institutions all had similar pressures on curriculum time and were equally dependent on informal or extra-curricular reforms. The article indicates that what could be read as a significant (inter)national movement, the ‘humanisation’ of medical education, was a variable process that resulted as much from individual initiative and social concerns as from intellectual currents and pedagogical theory.

These UK case studies build on the more extensive historiography of twentieth-century medical education in North America, including John Harley Warner’s relevant article on ‘The Humanising Power of Medical History’.^11^ The article also develops such work by considering the different ways in which ‘human’ aspects of medical education operated, particularly beyond the context of arts and humanities education. Despite a long tradition of medical ‘humanism’ being articulated through subjects such as medical history, students and educators sought the human aspects of medicine in a wide range of contexts in the late twentieth century. UK medical education echoed some trends taking place in the USA at this time, in which – to quote medical historian Brian Dolan – medical history no longer ‘stood alone to offer humanistic perspectives on medicine’ and new disciplines offered ‘alternative models for analysing cultural dynamics in medicine’.^12^ These ‘alternative models’ of the human aspects of medical education await in-depth study in the UK context.

## 1 Students and Society

In 1967 three educators at the University of Edinburgh wrote an article for *The Lancet* in which they noted:

There is a growing danger that the student may become so preoccupied with the minutiae of the specialties and with the technologies of the laboratory that he loses sight of the patient as a “whole person” functioning in a social context, and this has led some medical educators to search for a counterbalancing element to build into the medical curriculum.^13^

This comment neatly summarised concerns that were circulating widely in medical literature, about the loss and – in consequence – the apparent need to remedy deficiencies of the medical curriculum. The GMC had expressed concerns about the overloading of a fact-based curriculum since the 1860s, but did so increasingly in the late twentieth century with a post-war rise of technical knowledge and related student disillusionment.^14^ Atomic bombs and concentration camp experiments also highlighted the problems of divorcing biomedical science and technology from humanity, driving a medical and scientific ethics movement that was particularly compelling to medical students.^15^ As Rachel H. Ellaway notes, concerns about technology were nothing new in the late twentieth century, but reached a ‘critical level’ that fuelled ‘the tension between the technological and human aspects of medical education’.^16^ The wider social context of what Ronald A. Carson, in his work on the history of the relationship between medicine and the humanities, labels the ‘turbulent ‘60s’ also fed into an international literature that promoted reform of medical practice more generally.^17^ This literature tapped into anxieties about rapid technological changes, and a growing social emphasis on marginalised voices and liberation movements.

In the 1960s and early 1970s many medical students and intellectuals, as well as patients themselves, called for the human aspects of medicine to be placed at the centre of medical practice.^18^ In the context of medical education, in addition to a general interest in patient-centred care, they focused on two key issues which are considered below in turn: firstly, the responsibility of medicine as a profession to engage with moral questions about human rights and scientific/medical ethics; secondly, the relationship between health and human behaviour through engagement with social and community medicine (not necessarily in the form of social sciences, but through a broader emphasis on the value of primary care for doctors’ relationships with communities and understandings of their health). In both of these, the human aspects of health care were moral and social obligations for the profession as a whole, rather than a skill or ‘competency’ of the individual doctor.

The palliative care movement in particular, which Cicely Saunders pioneered in the UK in the 1960s, posed specific types of moral ethical questions that appealed to some medical students.^19^ The London Medical Group (LMG) was key in introducing medical ethics to undergraduate education in an informal way, with a ‘medical group’ format that later was taken up in other major cities. LMG was, in its own words, a free ‘non-partisan student group for the study of issues raised by the practice of medicine which concern other disciplines… lectures and symposia are addressed to medical, nursing and other students in the twelve London teaching hospitals’.^20^ Attendance was voluntary, but there was an average of seventy-six students in attendance for lectures in 1974.^21^ Although a small and informal group, the LMG is significant for its particular interest in medical ethics and the human aspects of medicine. Saunders gave one of the earliest lectures to the LMG on terminal pain, and continued to do so at the request of students for the following twenty-five years in a lecture that – in the words of Edward Shotter – ‘became a classic’.^22^ Saunders later noted that:

[T]heir repeated request for the topic illustrates how the students continued to demand a look at the humanistic side of medical education. That the particular subject of end of life care is a challenging way of approaching this is illustrated by the number of Medical Groups in other cities that chose it as their inaugural lecture.^23^

The interest of London students – and soon that of others in cities around the country – in the topic of pain and the ethics of pain management was, in this context, a way into the human aspects of medicine rather a *specific* interest in the topic of pain. Critiques of biomechanical models of pain connected to wider questions about holistic health care, often thought of as part of what Saunders called the ‘humanistic side of medical education’. Other themes of LMG conferences reinforce Saunders’s point, illustrating a particular interest from the late 1960s to 1980s in topics of social or moral significance and human rights such as: the prolongation of life (1967); the quality of life (1971); iatrogenic disease (1975); human rights in medicine (1983) and children at risk (1989). Although medical groups focused on ethics, the early remit of medical ethics education was broad, multidisciplinary and often related to subjects that fell under the broad umbrella of putting the ‘human’ at the centre of medical care.

Students in other institutions even gained some direct input into curriculum design through their work in medical groups. Building on the success of the Manchester Medical Group (an extension of the London format), for example, the Curriculum Committee at the University of Manchester included student representatives throughout the period of reform. In 1982 the committee included four student representatives who expressed an interest in further lectures and seminars on medical ethics.^24^ The students wrote a report on the matter, leading the committee to promise ‘renewed emphasis’ on ethical questions within existing courses and a review of such arrangements with the intention of formalising them. It seems likely that student representatives played at least some role in shaping the committee’s gradual shift towards providing curriculum space for the human aspects of medicine. In the late 1980s curriculum committees in Manchester began explicitly to recommend tutorials to deal with the ‘humanitarian’ aspects of medicine.

Students were not alone in promoting such subjects, but seemingly had more success than others: it was not until the 1980s that medical ethics gained a formal and widespread foothold in medical schools. In 1957 the GMC had recommended the introduction of ethics teaching, but had given medical schools autonomy instead of strict guidelines on how to do so. Professor and ethicist Roger Higgs also recalls that ‘professional conservatism was strong’, when ethics courses began to emerge in the 1960s.^25^ Some contemporaries and professional bodies rejected critiques of medical care, which formed the basis of many calls for change and for better engagement with ethics. They claimed that most general practitioners already gave – in the words of one physician writing on medical education in the *British Medical Journal* in 1965 – ‘first-class all-round care to their patients’.^26^ This resistance indicates that not everybody supported calls for more engagement with the human aspects of medicine and medical education, with one argument being that medical practitioners *already* engaged with human rights and delivered patient-centred care. Undoubtedly there were comparable students, with little interest in change. However, those students who did support reform seemingly had more success in achieving practical change than staff – even if only, at first, in an informal and extra-curricular capacity.

The story of social medicine, the study of the social dimensions of health and illness, is very similar to that of medical ethics.^27^ It shows the slow nature of formal changes alongside students’ growing interest in so-called ‘human’ questions. In the UK, the requirements of the NHS were particularly conducive to a social model of health care and – by extension – health care education. Social medicine had been on the agenda of medical schools since the 1940s and was explicitly promoted in the 1944 Goodenough Report, the report of the government’s Inter-Departmental Committee on Medical Schools.^28^ The 1968 ‘Todd Report’ of the Royal Commission on Medical Education (1965–8) emphasised that ‘the student should learn about man in all his aspects not only as a patient but also as a social being’.^29^ These claims were inextricably bound with questions of patient-centred care and the human aspects of medicine, including the human and social causes of illness. As Dean of the Faculty of Medicine at the University of Birmingham, Dr Douglas Hubble, commented in a *British Medical Journal* article in 1966, writing on ‘humanism in medicine’, the British doctor was an ‘agent of society’ and the doctor–patient relationship was part of ‘our social organisation’.^30^ Within this framework, ‘humanism’ revolved around a more egalitarian doctor–patient relationship at a time when social inequality in general was being challenged. It differed from the explicit focus on human rights within ethics, but was grounded in a similar moral philosophy.

Significant new intellectual works fuelled the growing interest in social dimensions of health care and the community role of the doctor, and in embedding such approaches within medical education. Most critiques of medicine at this time, however, came from outside the medical profession. Philosopher and priest Ivan Illich’s *Medical Nemesis* (1975), for example, examined the iatrogenic impact of an overly medicalised society. Two years later, *Science* printed George Engel’s influential critique of reductionist biomedicine that left ‘no room within its framework for the social, psychological, and behavioural dimensions of illness’.^31^ Emphasising the wider social and moral dimensions of the doctor–patient relationship, in a way that connected with the late twentieth-century rise of medical sociology and interest in the ‘sick role’, Engel suggested that a ‘Biopsychosocial Model’ of clinical care would provide more opportunities for engaging with the holistic and human aspects of medicine.^32^ Engel viewed medical education as a key site for remedying the apparent deficiencies of the ‘biomedical model’, writing that ‘[t]he average physician today completes his formal education with impressive capabilities to deal with the more technical aspects of bodily disease, yet when it comes to dealing with the human side of illness and patient care he displays little more than the native ability and personal qualities with which he entered medical school’.^33^ In all of these frameworks, medicine, illness and recovery were as much social as individual or biological phenomena.

Despite the promotion of social medicine, both in terms of engagement with the social dimensions of illness and a wider interest in the doctor’s community role, Jane Lewis notes that ‘within the medical schools social medicine made little headway’ and ‘most schools reacted only by slightly modifying their departments of public health’.^34^ Such claims are supported by accounts of the 1967 Annual Meeting of the Royal College of General Practitioners in London, which noted that King’s College London sought to turn towards community medicine but was hampered by limited budgets.^35^ However, students showed ongoing engagement with social questions. The LMG conference on iatrogenic disease took place in the same year that Illich’s book on the subject was published.^36^ Many key figures in UK medical education also engaged with such literature in their formative student years and went on to play roles in ‘humanistic’ fields. In a recent Wellcome Trust Witness seminar, for example, Kenneth C. Calman recalled the widespread influence of a number of important texts that raised moral questions about medical care. These included the work of Illich, as well as of others such as René Dubos (1959), Henry Miller (1973) and Thomas McKeown (1976).^37^

Students engaged particularly with the work of English novelist John Berger. Berger’s seminal text *A Fortunate Man* (1967) about a country doctor drew upon Michael Balint’s psychologically informed work and dealt with the importance of human relationships and patient-orientated health care.^38^ Although *A Fortunate Man* related to individual doctor-patient relationships and psychology, this book seemingly also seemingly struck a particular chord with students for its representation of human relationships at a social level. Professor Gene Feder, who qualified from Guy’s Hospital Medical School, recalled ‘reading [*A Fortunate Man*] as a medical student and thinking: this is why I want to be a doctor’ in the late 1970s.^39^ In a recent interview Professor Brian Hurwitz of King’s College London, a pioneer of education in narrative medicine in the UK, also remembered reading *A Fortunate Man* soon after its release while training in Cambridge as a book that stood ‘for the human in a relatively dehumanised health-care context’.^40^ He notes that, like many contemporaries, he read *A Fortunate Man* because of Berger’s role as a left-wing ‘iconoclast’ and had a particular interest in the book’s integration of social approaches such as Marxism, psychoanalysis and literary theory.^41^ Such social and intellectual trends help to explain not only the nature of these texts, but their positive reception.

It is significant that many of these educators recall the influence of such texts on their thinking as students, rather than as qualified practitioners, in line with the general trend for students to be most receptive to such liberal thought. The work seemingly tapped into a liberal zeitgeist of the late twentieth century and spoke to students’ interest in the general place of doctors in society, as well as in the individual clinical encounter. Medical students thus demonstrated an interest in social medicine in its broadest terms, echoing their approach to medical ethics, in which social medicine was a form of moral contract between the doctor as community member and his patients as a collective. In this framework social medicine included the social dimensions of illness, but was not necessarily – or purely – a social science.

Students and some individual staff members were vocal about their dissatisfaction with conservative medical curricula throughout this period. In 1978, a Manchester-based working party issued the Howat Report on medical examinations that still showed few signs of concern about the human aspects of medicine or patient-centredness. This report was based on the assumption that a primary goal of medical education was ‘to produce a medically oriented scientist’.^42^ The only skills mentioned in the report were ‘history-taking, physical examination, case reporting’, rather than any form of relationship-building with individuals or communities.^43^ The Howat Report, however, was not universally accepted by those who sought more forward-looking curricula or by students seeking to promote a more holistic model of health care. One neurology lecturer and member of the working party acknowledged that the report ‘has created some interest among students… some are alarmed by its apparently retrogressive recommendations’.^44^ Although those students failed to gain the forward-thinking curriculum that they apparently desired, they certainly made their discontent clear and laid the groundwork for changes that would come later, in the 1980s. The lecturer defended the suggestions of 1978 as an attempt to reverse reforms of the 1960s based on ‘administrative dreams’, in which all education was formulated as an ‘internally consistent body of knowledge’ and projected onto passive students.^45^ This passivity was seemingly indeed a ‘dream’ by the 1970s.

This is not to say that there was no change at all in medical schools. The growth of primary care as a specific area of education and study was a particularly important shift. It connected to the social questions in which students showed an interest (the general practitioner as a member of a community) and the individual doctor–patient relationships with which educators (later) appeared more concerned. Remembering his own medical practice in the UK and the history of general practice, Roger Higgs notes that the traditional paternalistic doctor–patient relationship began to be questioned in the 1960s because ‘relationships in general were under the microscope’.^46^ Higgs here referred to some of the same broad social questions about relationships and authority that fuelled the concerns of writers such as Illich and Engel. Individuals like Higgs played a role in translating such movements into a form relevant to medical education, by increasing the profile of general practice in medical schools over the course of the late twentieth century. Higgs described the King’s College London (KCL) Department of General Practice Studies as still ‘embryonic’ in 1978 but it was part of an important wider movement, in which the number of departments of general practice in the UK grew from one in 1965 to eleven in 1972.^47^ Even within general practice, however, early changes to medical curricula were pushed forward by individuals and were limited in scope and success.

In his history of the medical school at London Hospital Medical College (LHMC), its former dean John Ellis wrote that the 1970s curriculum was not guided by a deliberate policy shift but that ‘comparison of the notes written by students and house officers in the seventies with those written by their predecessors of forty years earlier reveals infinitely more recorded about the patient as a person [in the 1970s]’.^48^ He attributed this in part to a shift in the type of diseases, to chronic conditions that allowed students to spend more time with patients, and this analysis has shown further the influence of intellectual and social trends on patient-centred medicine and education. Ellis’s comments support medical school records that suggest a growing interest in engagement with the patient as a ‘whole person’ in the post-war period, driven in part by changes in students’ attitudes and culture.

These attitudinal shifts in UK institutions echoed those across the Atlantic, and were part of wider social trends. Kenneth Ludmerer notes that US students similarly, as a category of ‘student activism’ that overlapped with protests and the promotion of educational reform, sought ‘to make the medical school more responsive to the health needs of the community’ in part because of ‘impatience with the requirement that they become adequately acquainted with the basic sciences before being allowed to participate in the study and care of patients’.^49^ The role of students in promoting the human aspects of health care was part of a wider culture shift within UK and US medical schools alike, in which students gained (or at least demanded) a greater voice. The medical schools considered here echoed a wider pattern of 1960s and 1970s student activism, albeit through socially orientated reform rather than the more widely known political protest, that focused on marginalised groups (in this case the patient) and on tackling inequality. Medical students had long ‘voted with their feet’ and shaped medical curricula implicitly through course selection long before the late twentieth century, including at the medical schools considered in this article, but they demonstrated a new engagement with intellectual trends and activeness in reforming ‘from below’.^50^

Overall the influence of students on reform, albeit in a piecemeal and informal fashion, supports Sam Leinster’s observations that medical students’ dissatisfaction with the educational system was a key driver of change in the late twentieth century.^51^ The rise of the consumer model of health care and medical education meant that students had a growing voice. Student activism connected to a growing number of patient groups that also promoted more patient-centred approaches to childbirth and resisted medical authority.^52^ As Jonathan Reinarz and Rebecca Wynter have observed, the consumer movement of the late 1960s complemented a wider convergence of Left and Right that critiqued medicine.^53^ The British Women’s Health Movement resisted the masculine culture of health care, at the same time as there was a growing number of female medical students to promote change ‘from within’ – even though they were still in the minority.^54^ This general social background fuelled an interest in patient-centred health care and in democratic forms of health care, based upon equal doctor–patient relationships at both individual and community levels. This background is crucial for understanding students’ interest in humanity as a collective entity.

Although it is clear that students had a particular interest in the ‘human’ as collective entity and as social being in the post-war period, the human aspects of medical education remained a confused and multifaceted concept in the 1970s. Students showed interest in a range of ‘big’ questions related to patient-centred and holistic care, human rights, the ethics of health care practices and the role of the doctor within communities. Some of these overlapped with individual doctor–patient relationships, such as questions about the ‘good death’, biopsychosocial models and general practice, but were often moral in nature and linked to broader liberal principles. It would not be until the 1980s and 1990s, when the locus of change shifted to ‘above’ or ‘within’ the medical school, that reform began to focus on particular educational models of the ‘human’ that connected to skills or training.

## 2 Communication and Competencies

The human aspects of medical education began more clearly to affect medical schools’ policies in the final decades of the twentieth century. The social and ethical aspects of medicine gained footholds in UK medical schools at the same time, but not in exactly the same form advocated by students. Ethics teaching narrowed towards the subject of ‘bioethics’, which differed slightly from students’ engagement with wide-ranging moral and social questions, while social medicine as a subject related more to social science than to students’ broader interest in the doctor’s social role and holistic models of health care.^55^ When approached ‘from within’ the medical school, the general practitioner became the focus of efforts to engage with the human aspects of medical education. This attention took two main forms: firstly, through education in communication skills and human relationships in the clinic; secondly, through attention to the individual attitudes and humanity of doctors. Although these trends apparently differed from students’ interests, the groundwork for a turn towards general practice as a focus of the human aspects of medicine had been laid many years earlier. General practice brought together social and individual models of patient-centred and holistic care.

Education in competencies and communication skills, most of which began to be introduced formally in the 1980s, cannot be understood without looking back to the post-war groundswell of interest in doctor–patient relationships. This background indicates that communication skills were not merely conceptualised as diagnostic tools but as part of relationship-building and the human aspects of health care. Roy Porter notes that patient movements made many medical educators and education theorists ‘aw[a]ke to patient dissatisfaction with the medical profession, the gripe that doctors were obtuse or authoritarian’.^56^ Staff, often those outside key positions of power, showed an interest in such questions within medical school magazines throughout the 1950s and 1960s. Although aimed at a student readership, *Zodiac*: *Journal* *of the* *Aberdeen University* *Medical Society* included multiple articles by members of staff who gave opinions on medical practice and education. In 1957, for example, the Aberdeen physician W. D. H. Conacher wrote in *Zodiac* that the aim of undergraduate medical education was not ‘merely the acquisition of technical knowledge …but a wider comprehension of the relationship of patient and doctor which can seldom be realized in the wards of a teaching hospital’.^57^ Conacher was not alone in emphasising the importance of human relationships to medical care in *Zodiac*. In 1960 an Aberdeen Professor of Mental Health noted ‘[i]f medicine is a field of learning, it is also a special form of human relationships …[I]t has always been recognised that the best doctors are those who have retained their sensitivity in these matters’.^58^

The *Manchester University Medical School Gazette* contained articles that articulated similar sentiments. As early as 1958, it published correspondence on ‘Education for a Changing Profession’ stating that the general practitioner should be ‘concerned primarily with the patient and not the disease’.^59^ The *Gazette* also published an article by John Ellis, then sub-dean of LHMC, calling for the reform of medical education in order to give the doctor a ‘scientific attitude of mind… while maintaining… the most humanitarian concern for the patient as a person’.^60^ The language of ‘humanitarianism’ brought together individualistic models of doctor–patient relations and the concept of a wider social agenda, being a term linked most commonly to welfare. It is significant that many of these articles located general practice education as a key site for reform, laying the groundwork for changes that would come later. Many of these also differed subtly from students in their approach to the human aspects of medicine. They focused on ‘human relationships’ at an individual level as a counterbalance to perceived fact-overload and on doctors’ ‘attitudes’ and ‘sensitivity’. Within such literature the ‘relationship’ was the individual doctor–patient relationship and ‘humanitarian’ approaches related to the ways in which doctors viewed and engaged with their patients.

Such publications did not directly inform curriculum change, nor did they expect to. In the early 1970s contributors to *Zodiac* recognised that it would be ‘naïve’ to expect rapid changes in ‘conservative’ British medical schools, but still argued that they should learn from the ‘adventurous approach to problems in medical education’ overseas.^61^ Such publications are best viewed as part of networks of idea exchange within universities, rather than as drivers of change. The editors of the *Manchester University Medical School Gazette* noted in 1960 that ‘our graduate circulation well exceeds the undergraduate circulation’.^62^ *Zodiac* had a circulation of 1000 in 1973.^63^ These gazettes, therefore, though only small, were significant parts of medical school culture, even if not a direct influence on curriculum reform. The articles cited here were also individual opinions rather than the general tone of the medical school literature, which included many articles that were scientific in focus. However, they represent important early examples of a shift towards interest in educating for better doctor–patient relationships. They also show how medical school literature, as a site of interaction between supportive staff and reformist students, could operate as one pressure for change.

A few members of staff did manage to achieve change in the post-war period, or at least to raise the profile of debates about the human aspects of medical education, but they were exceptional rather than representative cases. John Ellis of LHMC was particularly vociferous in this respect, and was in a position of international influence. He published on such subjects as ‘Human Values in Medical Education’ in the 1970s and promoted the teaching of communication skills internationally as part of his role as secretary for the Association for the Study of Medical Education (ASME); ASME also played a key role in investigating and sharing details of international efforts to ‘humanise’ medical education.^64^ Ellis himself was also part of international professional networks interested specifically in questions of the human aspects of medical education. In the 1960s he was, for example, on familiar terms and in regular correspondence with George T. Harrell of Penn State University, who was another advocate of ‘humanistic’ medical education.^65^

Ellis’s particular interest in doctor–patient communication anticipated a later trend, within medical schools, to focus on communication skills as a way into the human aspects of health care. In the 1970s the GMC issued new draft recommendations to medical schools that included explicit references to the value of teaching communication skills, for example, rather than the earlier liberalisation and vague guidance away from fact-based education.^66^ In practice some courses in communication or consultation skills still constituted fairly traditional history taking. Their appeal also lay in part in the fact that communication skills had an instrumental value (diagnosis) and could be measured or tested (through the new Objective Structured Clinical Examinations or OSCES).^67^ However, it would be overly reductionist to view communication skills as *purely* instrumental in purpose. Jo Brown notes that the rise of the consumer model within the NHS contributed to ‘the development of a new and more egalitarian relationship between doctor and patient’ and to a shift in the nature of communication in the clinical encounter.^68^ Medical school prospectuses also discussed doctor–patient communication and relationships in ‘humanitarian’ terms rather than purely as diagnostic tools. In 1984, for example, the KCL medical school’s prospectus newly stated that ‘[g]reat emphasis is placed on the student’s attitude to and concern for his patients’.^69^

Education in ‘competencies’ can be viewed as a continuation of earlier trends in the human aspects of medical education, rather than necessarily a break from them. Training in communication skills focused increasingly on one-to-one encounters between doctor and patient, but built upon, for example, new understandings of how to ‘break bad news’, holistic health care and a more ‘egalitarian’ social model. Although the ‘human’ in this situation was increasingly an individual patient, and formal educational initiatives involved doctors being taught measurable ‘competencies’ rather than promoting general moral or social principles for health care, there was certainly some overlap in the principles underlying the different pedagogical trends. As David S. Jones *et al.* argue, ‘competencies have emerged as the guiding philosophy for the design of educational systems’, but should not be viewed as incompatible with ‘humanistic’ principles or disciplines.^70^

Although not gaining traction in medical schools until the 1980s, and in some places the 1990s, there were long roots to the decision to introduce communication skills into medical curricula. There were belated echoes of Michael Balint’s book title *The Doctor, his Patient and the Illness* (1957) in the LHMC prospectus of 1980, for example, which newly referred to educating students to ‘interpret, understand and treat the patient and his illness’.^71^ It is perhaps unsurprising, given Ellis’ role, that LHMC was the first of the medical schools considered here to include such a comment in its prospectus. LHMC ran ‘Consultation Skills’ courses by the 1980s, although correspondence between staff indicates that these were still set up by arrangement between individuals and medical firms rather than centrally directed.^72^ The LHMC prospectus’ implicit reference to Balint is significant. While medical students themselves had seemingly latched onto Berger’s *A Fortunate Man*, inspired by Balint but with a particular focus on local communities, medical schools drew more upon the aspects of Balint’s work that spoke to the skills and demeanour of individual doctors. Balint’s seminal text, *The Doctor, his Patient and the Illness*, advocated the use of psychology to improve the clinical interview and emphasised the value of training in attitudes and professionalism. Balint *et al.* also paid further attention to the challenges of attitudinal training for general practitioners in *A Study of Doctors* (1966), as part of a growing literature on individual medical students’ attitudes.^73^

The work of Balint raises another key human aspect of medical education, as indicated implicitly by the KCL and LHMC prospectuses: doctors’ ‘attitudes to’ patients. Growing concerns about the attitudes of medical practitioners, and by extension medical students, are illustrated by responses to US sociologist Renée Fox’s concept of ‘detached concern’. When Fox first conceptualised ‘detached concern’ in 1959 she did not intend for detachment to be emphasised *over* concern, but rather for the two to operate together in a dualistic manner.^74^ At first ‘detached concern’ was seemingly taken in this spirit, but Fox has recently complained that the concept was later picked up in ‘discussions about how the care of patients by health practitioners can be more effectively “humanised” ’.^75^ The *British Medical Journal* as early as 1965, commented that ‘this necessary “detached concern” which is conferred by medical education and experience has been viewed in some quarters as going too far, leading to a loss of idealism, an unfortunate callousness induced by professional training’.^76^ Like Flexner’s 1910 report, Fox’s work on ‘detached concern’ initially supported the goal of ‘humanising’ medicine but came later to be set up as a straw man, symbolic of ‘dehumanisation’.^77^ It seems that this dualism of ‘detachment’ and ‘concern’ underpinned philosophies of educational change: reform needed to counter a perceived problem (the loss of the ‘patient’ in the clinic and the decline of medical compassion) to be a worthwhile curriculum innovation. The KCL and LHMC prospectuses support this theory, with their new and explicit goal of educating for improved ‘attitudes’ and ‘understanding’ as well as skills: the doctor’s individual humanity thus began to be increasingly central to pedagogical discussions.

Despite evidence of shifts in educational philosophy at LHMC and KCL in the early 1980s, reform was still uneven and changes came slightly later elsewhere. Prospectuses from St Mary’s Hospital Medical School in Paddington indicate that it was relatively conservative throughout the 1980s.^78^ There was support for reform from a number of quarters including, according to one historian of the medical school, a dean who lamented how facts were ‘hurled at students like hand grenades’ and students who ‘resisted the scientization and specialization of the curriculum’.^79^ However, St Mary’s struggled to achieve practical change in a context of competing pressures and only overhauled the curriculum after the GMC’s publication of *Tomorrow’s Doctors* in 1993. In the mid-1980s the tone of the Aberdeen student prospectus on doctor–patient relations remained detached, despite the early emphasis on the value of humanistic medical education presented in *Zodiac*. In 1985, the prospectus stated that students were given ‘an introductory series of lectures on how to examine and question patients’ to prepare them for clinical work.^80^ The ‘human’ side of medicine was not clearly visible in this lecture format, except in its most instrumental form with an emphasis on ‘how to examine and question patients’ rather than on listening to and forming relationships with patients. However, in 1986 the prospectus added the following statement: ‘A vital component of a medical student’s education is learning how to communicate with patients, how to gain their confidence, and how to understand the psychological and social background to their physical problems’.^81^ Although it is difficult to assess the extent to which a prospectus represents a real change in practice, this shift in tone is significant. From a lecture on learning to ‘question’ patients, the emphasis moved to ‘communication *with* patients’ and gaining a real insight into their lives at both ‘psychological and social’ levels in order to better to understand physical symptoms. This framework bridged the individual and social models of humanism in health care, bringing in a version of the biopsychosocial model, albeit explicitly in the service of improved diagnosis.

In general, medical school records show a more formal interest in advocating and encouraging the human aspects of medicine from the 1980s onwards, but there was by no means a revolution in what educators labelled ‘humanistic’ or ‘humanitarian’ education. As late as 1988 a Manchester Working Party on medical education noted that ‘some individuals and some departments make notable contributions in exposing students to the humanitarian aspects of medicine’, but also observed that such contributions were unsystematic and therefore coverage was ‘inadequate’.^82^ Others at Manchester Medical School also continued to defend a science-based curriculum, for example by writing to the Working Party in defence of education in specialties.^83^ In the end this Working Party came to recommend a new tutorial system for medical students for the purpose of ‘exposing students to the humanitarian aspects of medicine’.^84^ Such tutorials were a forum in which students could discuss issues related to ethics and to communication skills both with patients and colleagues. Although the reason for the tutorial format was not recorded in committee minutes, it may be significant that such a system did not necessitate extensive curriculum time or reforming the undergraduate degree as a whole. The new tutorials represented a formal reform in line with the goal of more ‘humanistic’ health care, but remained a supplementary rather than core component of the curriculum.

In the final decades of the twentieth century medical schools increasingly advocated ‘humanitarian’, ‘humanism’ and ‘humanistic’ medicine. They used such terms often indistinguishably, but most commonly in relation to doctor–patient relationships, communication skills and doctors’ attitudes to patients. This focus differed slightly from that of medical students, whose interests had apparently been in medical ethics and the doctor’s role as a community member, but drew upon many of the same principles in advocating equality in the doctor–patient relationship alongside holistic and patient-centred primary care. Perhaps because ethics and social medicine were less problematic absences in the curriculum by the 1980s and 1990s, and because of the clearer instrumental value of skills that aided diagnosis, individual doctor–patient communication and relationships often became a focus of formal curriculum changes. Although many of these shifts were more ideological than practical, such new pedagogic principles aid an understanding of some of the key developments in modern medical education. The human aspects of medicine were connected in particular to the rise of competencies in medical education and were not necessarily incompatible with the instrumental aspects of diagnosis.

## 3 Conclusions

In 1980 a reader at the University of Edinburgh Medical School wrote in the *British Medical Journal* that ‘[t]he inflexible administrative structure of the traditional medical school has been likened to “balkanisation”…, constituting an insurmountable barrier to holistic and humanistic medicine’.^85^ This article has shown that extensive structural change was indeed rare, both within and beyond Scotland, but that – rather than barriers being entirely ‘insurmountable’ – ‘humanistic’ educational reform was achieved through informal and often individual initiative. Local responses to international and national trends in thinking about the human aspects of medicine were varied, but there were some notable trends in the late twentieth century. Perhaps most significantly, students were central to post-war engagement with human aspects of medicine as part of a wider interest in questions of social equality and the doctor’s community role. In this framework, engagement with the human aspects of medicine was a moral obligation and the patient could not be separated from society and community. The later turn to teaching communication skills and improving doctors’ attitudes, which was most visible in medical schools in the 1980s, took a more individual model of the doctor–patient relationship. However, this article has sought to show that these trends were not as separate as they first appear. Medical schools’ discussion of ‘humanitarian’ medicine indicated a wider welfare agenda, despite often referring to individual doctor–patient relationships. The general practitioner also came to be an important figure in the human aspects of health care and bridged moral, social and individual issues.

This article has shed light on the ways in which one educational principle fed into different trends in late twentieth-century medical education, and indicated the potential value of looking at the history of education through the lens of concepts rather than subjects. It shows a subtle turn to the ‘human’ in medical education, and the different ways in which this interest was expressed – and, in some contexts, resisted or ignored. Although it shows a growth in interest in the human aspects of medical education, its findings do not indicate any kind of uninterrupted rise of ‘humanistic’ medicine or medical education. The topic remains one of great debate. In the 1990s and 2000s, albeit beyond the scope of this study, the ‘humanistic’ agenda took new forms in the UK as part of the growing role of the arts and humanities in medical education.^86^ However, some scholars have called for a move away from or a more critical approach to the ‘humanistic’ as a framing device for educational interventions.^87^ In other contexts, concerns about the ‘dehumanisation’ of medicine are still widely articulated and solutions sought both within and beyond medical education. The Francis report, a public inquiry into the running of the Mid Staffordshire Foundation NHS Trust conducted between 2005 and 2009, provided evidence of the need to treat patients more as – in the words of the health secretary in 2013 – ‘human beings, and not as numbers’.^88^ Such reports demonstrate an ongoing concern about how to ‘(re)humanise’ medicine and the need for a more critical engagement with what we actually mean by the ‘human’ aspects of medical care, including the historical context.

## Footnotes

1.

*Punch, or the London Charivari*, 22 April 1914, 310.

2.
The 1953 report liberalised medical education, partly in response to the perceived requirements of the new National Health Service (NHS). This study ends in 1993, when local reform became less relevant as GMC’s *Tomorrow’s Doctors* (1993) formally introduced Special Subject Components (SSCs) that allowed undergraduate students to study ‘special subjects’ in the humanities.

3.
Some medical schools also used the term ‘aspects’, including early initiatives in the USA such as the Humanistic Aspects of Medical Education (HAME) program at NYU School of Medicine (1979).

4.
See George Sarton, ‘The New Humanism’, *Isis*, 6, 1 (1924), 11.

5.
Thomas Neville Bonner, ‘Abraham Flexner as Critic of British and Continental Medical Education’, *Medical History*, 33, 4 (1989), 472; Alan Bleakley, ‘Towards a “Critical Medical Humanities” ’, in Victoria Bates, Alan Bleakley and Sam Goodman (eds), *Medicine, Health and the Arts: Approaches to the Medical Humanities* (Abingdon; New York: Routledge, 2014), 18.10.1017/s0025727300049942PMC10359362682082

6.
Edmund D. Pellegrino, ‘The reconciliation of technology and humanism: A Flexnerian task 75 years late’, in Charles Vevier (ed.), *Flexner: 75 Years Later: A Current Commentary on Medical Education* (London; Lanham MD: University Press of America, 1987), 78.

7.
For example, Jonathan Reinarz, ‘Unearthing and Dissecting the Records of English Provincial Medical Education, *c* 1825–1948’, *Social History of Medicine*, 21, 2 (2008), 381–92.

8.
For example, Keir Waddington, *Medical Education at St. Bartholomew’s Hospital: 1123–1995* (Woodbridge: Boydell & Brewer, 2003); Dorothy Porter (ed.), *Social Medicine and Medical Sociology in the Twentieth Century* (Amsterdam: Rodopi, 1997).

9.
Undergraduate education is the focus here because of the GMC reports that frame the study (1957 and 1993) and the general emphasis on changing the basic training of *all*doctors within the literature on human aspects of medical care. However, reforms also took place in postgraduate training and continuing professional development at this time.

10.
On the difference between the development of London, Scotland and all other UK medical schools see Thomas Neville Bonner, *Becoming a Physician: Medical Education in Britain, France, Germany and the United States 1750–1945* (Baltimore, MD; London: Johns Hopkins University Press, 2000).

11.
John Harley Warner, ‘The Humanising Power of Medical History: Responses to Biomedicine in the 20th Century United States’, *Medical Humanities*, 37, 2 (2011), 91–6.10.1136/medhum-2011-01003421807650

12.
Brian Dolan, ‘Second Opinions: History, Medical Humanities and Medical Education’, *Social History of Medicine*, 23, 2 (2010), 396, 397.

13.
F. M. Martin, F. M. McPherson and P. R. Mayo, ‘A Course in Psychology and Sociology for Medical Students’, *The Lancet*, 19 August 1967, 41–13.10.1016/s0140-6736(67)92026-04166347

14.
Sam J. Leinster, ‘The history of change in the UK’, in Penelope Cavenagh, Sam J. Leinster and Susan Miles (eds), *The Changing Face of Medical Education* (Abingdon: Radcliffe Publishing, 2011), 3, 6.

15.
Such concerns were widely articulated in the UK and US in the post-war years, particularly in relation to the ethical aspects of medicine and science; see, for example, A. C. Ivy, ‘Some Ethical Implications of Science’, *School Science and Mathematics*, 48, 3 (1948), 206; Irving Ladimer, ‘Ethical and Legal Aspects of Medical Research on Human Beings’, *Journal of Public Law*, 3 (1954), 46–511; J. G. Thwaites, ‘Ethics and Medicine’, *British Medical Journal*, 24 May 1952, 1118.

16.
Rachel H. Ellaway, ‘Medical Education and the War with the Machines’, *Medical Teacher*, 36, 10 (2014), 917–18.10.3109/0142159X.2014.95508825216159

17.
Ronald A. Carson, ‘Engaged Humanities: Moral Work in the Precincts of Medicine’, *Perspectives in Biology and Medicine*, 50, 3 (2007), 321–33.10.1353/pbm.2007.002517660628

18.
For example, see Nancy Tomes, ‘The Patient as a Policy Factor: A Historical Case Study of the Consumer/Survivor Movement in Mental Health’, *Health Affairs*, 25, 3 (2006), 720–9.10.1377/hlthaff.25.3.72016684736

19.
David Clark, ‘From Margins to Centre: A Review of the History of Palliative Care in Cancer’, *The Lancet Oncology*, 8, 5 (2007), 430–8; David Clark and C. Von Gunten, ‘The development of palliative medicine in the UK and Ireland’, in Eduardo Bruera *et al.* (eds), *Textbook of Palliative Medicine* (Boca Raton, FL: Taylor Francis Group, 2006), 3–11.10.1016/S1470-2045(07)70138-917466900

20.
L.A. Reynolds and E.M. Tansey (eds), *Medical Ethics Education in Britain, 1963–1993*, Wellcome Witnesses to Twentieth-Century Medicine (London: Wellcome Trust Centre for the History of Medicine, 2006), xxii; on medical groups see also Wellcome Library, GC/253/a/31/8, Medical Ethics in Britain Related Documents, 1963–2006.

21.
Wellcome Library, GC/253/a/31/8, Medical Ethics in Britain.

22.
Reynolds and Tansey, *op. cit.* (note 20), 8.

23.

*Ibid*., 77.

24.
Archive of the Faculty of Medicine, The University of Manchester Library, GB 133 FME/3/2, Manchester Curriculum Committee, 1982.

25.
Roger Higgs, ‘The medical paradigm: Changing landscapes’, in Deborah Kirklin and Ruth Richardson (eds), *The Healing Environment: Without and Within* (London: Royal College of Physicians of London, 2003), 185.

26.
Alastair Hunter, ‘Medical Education and Medical Practice’, *British Medical Journal*, 4 September 1965, 556.

27.
This article uses ‘social medicine’ and ‘community medicine’ in line with the phrasing of contemporary sources, but in broad terms treats them as very similar concepts because they were often used interchangeably. See, for example, Milton Terris, ‘The Distinction between Public Health and Community/Social/Preventive Medicine’, *Journal of Public Health Policy*, 6, 4 (1985), 435–39.

28.
Nigel Oswald, ‘Training doctors for the National Health Service: Social medicine, medical education and the GMC 1936–48’, in Dorothy Porter, *Social Medicine*, *op. cit.* (note 8), 73.9459053

29.

*Report of the Royal Commission on Medical Education 1965*–*8* (London: HMSO, 1968), s. 293.

30.
Douglas Hubble, ‘Medicine Science, Society, and Human Values’, *British Medical Journal*, (1966), 475.10.1136/bmj.1.5485.474PMC18436225902688

31.
George L. Engel, ‘The Need for a New Medical Model: A Challenge for Biomedicine’, *Science*, 196, 4286 (1977), 129–36.10.1126/science.847460847460

32.
On the emergence of the ‘sick role’ in ‘medical sociology’ in the 1950s, see John C. Burnham, ‘The Death of the Sick Role’, *Social History of Medicine*, 25, 4 (2012), 761–6; on how the ‘sick role’ fed into contemporary literature see also Engel, ‘The Need for a New Medical Model’, 324.

33.
George L. Engel, ‘The Biopsychosocial Model and the Education of Health Professional’, *Annals of the New York Academy of Sciences*, 310, 1 (1978), 169.10.1111/j.1749-6632.1978.tb22070.x290321

34.
Jane Lewis, ‘Public health doctors and AIDS as a public health issue’, in Virginia Berridge and Philip Strong, *AIDS and Contemporary History* (Cambridge: Cambridge University Press, 2002), p. 47; Jane Lewis, ‘Providers, “consumers”, the state and the delivery of health care services in twentieth-century Britain’, in Andrew Wear, *Medicine in Society: Historical Essays* (Cambridge: Cambridge University Press, 1992), p. 336.

35.
‘Medicine, Science and Humanity – Annual Meeting of R.C.G.P.’, *British Medical Journal*, 2 December 1967, 546.10.1136/bmj.4.5578.546PMC17491126065998

36.
Ivan Illich, *Medical Nemesis: the Expropriation of Health* (London: Calder & Boyars Ltd, 1975).

37.
Reynolds and Tansey, *op. cit.* (note 20), xxi.

38.
Anne Whitehead, ‘The medical humanities: A literary perspective’, in Bates, Bleakley and Goodman, *op. cit.* (note 5), 110.

39.
Gene Feder, ‘A Fortunate Man: Still The Most Important Book about General Practice Ever Written’, *British Journal of General Practice*, 55, 512 (2005), 246–7.

40.
John D. Engel, Lura L. Pethtel and Joseph Zarconi, *Developing Clinicians’ Career Pathways in Narrative and Relationship-Centered Care: Footprints of Clinician Pioneers* (London: Radcliffe, 2012), 131, 139.

41.
Personal correspondence with the author, with thanks to Professor Brian Hurwitz for giving permission to cite his answer to this question.

42.
Archive of the Faculty of Medicine, The University of Manchester Library, ‘The Howat Report on Undergraduate Medical Education’, *Medical Gazette*, 57, 2 (1978), 32.

43.

*Ibid*., 32.

44.

*Ibid*., 33.

45.

*Ibid*., 34.

46.
Higgs, *op. cit.* (note 25), 183.

47.
Roger Higgs, ‘CABGs and KINGS: Relevance and Realism in the Teaching of Clinical Ethics in Camberwell’, *Journal of Medical Ethics*, 13, 3 (1987), 157; Patrick Sarsfield Byrne, ‘University Departments of General Practice and the Undergraduate Teaching of General Practice in the United Kingdom in 1972’, *The Journal of the Royal College of General Practitioners*, 23, suppl. 1 (1973), 1–12.

48.
John Ellis, *LHMC, 1785*–*1985: The Story of the London Hospital Medical College, England’s First Medical School* (London: LHMC, 1986), 163.

49.
Kenneth M. Ludmerer, *Time to Heal: American Medical Education from the Turn of the Century to the Era of Managed Care* (Oxford: Oxford University Press, 1999) 239.

50.
Jonathan Reinarz and Rebecca Wynter, ‘Introduction: Towards a history of complaining about medicine’, in Jonathan Reinarz and Rebecca Wynter (eds), *Complaints, Controversies and Grievances in Medicine: Historical and Social Science Perspectives* (Abingdon; New York: Routledge, 2014), 10; Stella V. F. Butler, ‘A Transformation in Training: The Formation of University Medical Faculties in Manchester, Leeds, and Liverpool, 187–84’ *Medical History*, 30, 2 (1986), 115–32.10.1017/s0025727300045348PMC11396133517521

51.
Leinster, *op. cit.* (note 14), 6.

52.
Charlotte Williamson, *Towards the Emancipation of Patients: Patients’ Experiences and the Patient Movement* (Bristol and Portland, OR: The Policy Press, 2010), 41. In relation to the history of drugs use, Alex Mold and Virginia Berridge have also shown that the rise of the patient service ‘user’ in the 1960s re-shaped the doctor–patient relationship, see Alex Mold and Virginia Berridge, ‘ “The Rise of the User?” Voluntary Organizations, the State and Illegal Drugs in England Since the 1960s’, *Drugs: Education, Prevention, and Policy*, 15, 5 (2008), 451–61; Uffe Juul Jensen and Gavin Mooney, ‘Introduction’, in Uffe Juul Jensen and Gavin Mooney (eds), *Changing Values in Medical and Healthcare Decision-Making* (Chichester: Wiley, 1990), 4.

53.
Reinarz and Wynter, *op. cit.* (note 50), 13.

54.
Although not as organised in the 1950s and 1960s as they would later become, there were already early developments in gendered patient movements at this time; ‘Women’s Health Network: State of the Art, Concepts, Approaches, Organizations in the Women’s Health Movement. Country Report: Great Britain’ (1998) <http://www.gesundheit-nds.de/ewhnet/Country_Reports/GB_E.pdf> (accessed 29 August 2014).

55.
On the difference between the LMG’s concept of ethics and ‘bioethics’ see, for example, Michael Whong-Barr, ‘Clinical Ethics Teaching in Britain: A History of the London Medical Group’ *New Review of Bioethics*, 1, 1 (2003), 73–84.10.1080/174002803200013143115706670

56.
Roy Porter, ‘The Patient’s View’, *Theory and Society*, 14, 2 (1985), 184.11618212

57.
W. D. H. Conacher, ‘Training for General Practice’, *Zodiac*, 4 (1957), 12–13.

58.
W. Malcolm Millar, ‘On Learning Medicine’, *Zodiac*, 7 (1960), 52.

59.
‘Letters to the Editor’, *Manchester University Medical School Gazette*, 38, 1 (1958), 30–1.

60.
John Ellis, ‘Preparation for Medicine as a Profession’, *Manchester University Medical School Gazette*, 40, 1 (1960), 3.

61.
‘Education in the Future Tense’, *Zodiac*, 21 (1973), 12–13.

62.
‘Editorial Comment’, *Manchester University Medical School Gazette*, 40, 1 (1960), 1.

63.

*Zodiac*, 21 (1973).

64.
John R. Ellis, ‘Human Values in Medical Education’, *Annals of Internal Medicine*, 85, 5 (1976), 659–68; The National Archives (TNA), Kew, ED 129/16, Information from ASME on Medical Education in Scandinavia, North America and Russia, 1966.10.7326/0003-4819-85-5-659984621

65.
Their correspondence from the late 1960s indicates both a personal familiarity and close work connections, through attendance at events and Ellis’s role as secretary of ASME. Penn State University Archives (PSUA), Pennsylvania, letter from John Ellis to George T. Harrell, 10 July 1967, SG3/5/60; PSUA, letter from John Ellis to George T. Harrell, 21 October 1966, SG3/5/60; PSUA, letter from John Ellis to George T. Harrell, 3 July 1968, SG3/5/60; PSUA, letter from George T. Harrell to John Ellis, 30 July 1968, SG3/5/60; PSUA, letter from George T. Harrell to John Ellis, 21 September 1966, SG3/5/60; PSUA, letter from John Ellis to George T. Harrell, 14 Sep. 1966, Harrell Papers SG3/5/60.

66.
Archive of the Faculty of Medicine, University of Manchester Library, GB 133 FME/3/2, Manchester Curriculum Committee, 1979.

67.
R. McG. Harden, *et al.*, ‘Assessment of Clinical Competence Using Objective Structured Examinations’, *British Medical Journal*, 22 February 1975, 447–51.10.1136/bmj.1.5955.447PMC16724231115966

68.
Jo Brown, ‘How Clinical Communication has become a Core Part of Medical Education in the UK’ *Medical Education*, 42, 3 (2008), 271–8.10.1111/j.1365-2923.2007.02955.x18275414

69.
King’s College London Archives, King’s College School of Medicine and Dentistry Prospectus 1984–5, 9, 14.

70.
David S. Jones, *et al.*, ‘Making the Case for History in Medical Education’, *Journal of the History of Medicine and Allied Sciences*, 70, 4 (2015), 623–52.10.1093/jhmas/jru02625395574

71.
Royal London Hospital Archives, London Hospital Medical College Prospectus 1980–1, 36.

72.
Royal London Hospital Archives, The London Hospital Medical College, Memorandum, 2 March 1984.

73.
M. Balint *et al.*, *A Study of Doctors: Mutual Selection and the Evaluation of Results in a Training Programme for Family Doctors* (London: Tavistock, 1966). International literature also started to address questions about students’ attitudes and empathy; see, for example, Howard S. Becker *et al.*, *Boys in White: Student Culture in Medical School* (Chicago, IL: Chicago University Press, 1961).

74.
Wendy Cadge and Clare Hammonds, ‘Reconsidering Detached Concern: The Case of Intensive-Care Nurses’, *Perspectives in Biology and Medicine*, 55, 2 (2012), 266–82; see also Robert K. Merton, George Reader and Patricia L. Kendall, *The Student-Physician: Introductory Studies in the Sociology of Medical Education* (Cambridge, MA: Harvard University Press, 1957).10.1353/pbm.2012.002122643763

75.
Fox cited in Cadge and Hammonds, *op. cit.* (note 74), 266.

76.
‘A Good Doctor’, *British Medical Journal*, 7 August 1965, 315.

77.
Jodi Halpern, ‘From Idealized Clinical Empathy to Empathic Communication in Medical Care’, *Medicine, Health Care and Philosophy*, 17, 2 (2014), 301–11.10.1007/s11019-013-9510-424343367

78.
St Mary’s Hospital Archives, St Mary’s Hospital Medical School Prospectuses for BSc Courses 1976–97.

79.
E.A. Heaman, *St Mary’s: The History of a London Teaching Hospital* (Montreal: McGill University Press, 2003), 327.

80.
University of Aberdeen, Special Collections, University of Aberdeen Undergraduate Prospectus for Entry in 1985, 106.

81.
University of Aberdeen, Special Collections, University of Aberdeen Undergraduate Prospectus for Entry in 1986, 100.

82.
Archive of the Faculty of Medicine, The University of Manchester Library, Clinical Curriculum Working Party 1986–9, FME/6/7.

83.
Archive of the Faculty of Medicine, Clinical Curriculum Working Party.

84.
Manchester, Clinical Curriculum Working Party 1986–9, FME/6/7.

85.
Donald S. McLaren, ‘What to do about Basic Medical Science’, *British Medical Journal*, 12 July 1980, 171.10.1136/bmj.281.6233.171PMC171358620793145

86.
Curriculum initiatives using the arts and humanities in medical education can be traced at least back to the 1950s in the US; see Alan Bleakley and Therese Jones, ‘A timeline of the medical humanities’, in Bates, Bleakley and Goodman, *op. cit.* (note 5), 281–4. However, so-called ‘medical humanities’ courses – particularly those involving the arts – were rare in UK medical education until after the 1993 publication of *Tomorrow’s Doctors* <http://78.158.56.101/archive/prs/view.html/PrsDocuments/95.html>(accessed 29 August 2014). On how ‘humanistic’ ideals have underpinned the medical humanities see Claire Hooker and Estelle Noonan, ‘Medical Humanities as Expressive of Western Culture’, *Medical Humanities*, 37, 2 (2011), 79–84.10.1136/medhum-2011-01012022114347

87.
Jane Macnaughton, for example, warns against making ‘uncritical’ links between arts, humanities and the ‘humane’; Jane Macnaughton, ‘Medical Humanities’ Challenge to Medicine’, *Journal of Evaluation in Clinical Practice*, 17, 5 (2011), 927–32. On the dangers of assuming that arts and humanities are inherently ‘humanising’ while technology and biomedicine are inherently ‘dehumanising’ see also Therese Jones, “ ‘Oh the humanities!”: Dissent, democracy and danger’, in Bates, Bleakley and Goodman, *op. cit.* (note 5).10.1111/j.1365-2753.2011.01728.xPMC443973721851510

88.
‘Mid Staffs report to spark NHS “culture change” ’, *Nursing Times*<http://www.nursingtimes.net/nursing-practice/specialisms/management/mid-staffs-report-to-spark-nhs-culture-change/5053324.article> (accessed: 27 October 2015).

